# The Expanding Phenotypical Spectrum of *WARS2*-Related Disorder: Four Novel Cases with a Common Recurrent Variant

**DOI:** 10.3390/genes14040822

**Published:** 2023-03-29

**Authors:** Martje G. Pauly, G. Christoph Korenke, Sokhna Haissatou Diaw, Anne Grözinger, Ana Cazurro-Gutiérrez, Belén Pérez-Dueñas, Victoria González, Alfons Macaya, Ana Teresa Serrano Antón, Borut Peterlin, Ivana Babić Božović, Aleš Maver, Alexander Münchau, Katja Lohmann

**Affiliations:** 1 Institute of Neurogenetics, University of Luebeck, Ratzeburger Allee 160, 23562 Luebeck, Germany; martje.pauly@neuro.uni-luebeck.de (M.G.P.); sokhna-aida.diaw@neuro.uni-luebeck.de (S.H.D.); anne.groezinger@gmx.de (A.G.); 2 Institute of Systems Motor Science, University of Luebeck, 23562 Luebeck, Germany; alexander.muenchau@neuro.uni-luebeck.de; 3 Department of Neurology, University Hospital Schleswig Holstein, 23562 Luebeck, Germany; 4 Department of Neuropediatrics, University Children’s Hospital, Klinikum Oldenburg, 26133 Oldenburg, Germany; korenke.christoph@klinikum-oldenburg.de; 5 Pediatric Neurology Research Group, Autonomous University of Barcelona, Hospital Universitari Vall d’Hebron, 08035 Barcelona, Spain; ana.cazurro@vhir.org (A.C.-G.); belen.perez@vhir.org (B.P.-D.); amacaya@vhebron.net (A.M.); 6 Center for Biomedical Network Research on Rare Diseases (CIBERER), 08035 Barcelona, Spain; 7 Department of Neurology, Autonomous University of Barcelona, Hospital Universitari Vall d’Hebron, 08035 Barcelona, Spain; mariavictoria.gonzalez@vallhebron.cat; 8 Clinical Genetic Section, Pediatric Service, Hospital Clinico Universitario Virgen de la Arrixaca, 30120 Murcia, Spain; anateserrano@gmail.com; 9 Clinical Institute of Genomic Medicine, University Medical Centre Ljubljana, 1000 Ljubljana, Slovenia; borut.peterlin@kclj.si (B.P.); ivana.babic.bozovic@kclj.si (I.B.B.); ales.maver@kclj.si (A.M.)

**Keywords:** *WARS2*, tremor, parkinsonism, levodopa

## Abstract

Biallelic variants in the mitochondrial form of the tryptophanyl-tRNA synthetases (*WARS2*) can cause a neurodevelopmental disorder with movement disorders including early-onset tremor–parkinsonism syndrome. Here, we describe four new patients, who all presented at a young age with a tremor–parkinsonism syndrome and responded well to levodopa. All patients carry the same recurrent, hypomorphic missense variant (NM_015836.4: c.37T>G; p.Trp13Gly) either together with a previously described truncating variant (NM_015836.4: c.797Cdel; p.Pro266ArgfsTer10), a novel truncating variant (NM_015836.4: c.346C>T; p.Gln116Ter), a novel canonical splice site variant (NM_015836.4: c.349-1G>A), or a novel missense variant (NM_015836.4: c.475A>C, p.Thr159Pro). We investigated the mitochondrial function in patients and found increased levels of mitochondrially encoded cytochrome C Oxidase II as part of the mitochondrial respiratory chain as well as decreased mitochondrial integrity and branching. Finally, we conducted a literature review and here summarize the broad phenotypical spectrum of reported *WARS2*-related disorders. In conclusion, *WARS2*-related disorders are diagnostically challenging diseases due to the broad phenotypic spectrum and the disease relevance of a relatively common missense change that is often filtered out in a diagnostic setting since it occurs in ~0.5% of the general European population.

## 1. Introduction

The mitochondrial form of the tryptophanyl-tRNA synthetases (*WARS2*), which catalyzes the aminoacylation of tRNA(trp) with tryptophan [1], is one of the 14 mitochondrial aminoacyl-tRNA synthetases related to neurological disorders [2]. The first cases with biallelic pathogenic variants in *WARS2*, described in 2017, had a predominant neurodevelopmental phenotype with developmental delay and intellectual disability, with only mild movement disorders such as tremor (OMIM: #617710) [3,4,5]. Later, additional cases [6,7,8,9,10,11,12,13,14] with prominent movement disorders (OMIM: # 619738) such as tremor and parkinsonism with a good response to levodopa [6,12,13,14] were described with only mild or no intellectual disability. Functional studies in different biosamples of patients with biallelic variants showed impaired or normal enzyme activity and protein levels of the mitochondrial respiratory chain depending on the type of investigated biomaterial and the specific variants [4,5,6,7,8,12].

Here, we describe four new patients with early-onset, levodopa-responsive tremor–parkinsonism syndromes with a common recurrent *WARS2* missense variant on one allele and an additional truncating or missense variant on the other allele.

## 2. Materials and Methods

### 2.1. Identification of Variants and Additional Patients

Our index patient was referred to our center because of an early-onset tremor–parkinsonism syndrome. The trio exome sequencing of P1 and the healthy parents was performed in 2018 (Agilent SureSelect Human All Exon V6 (Illumina, Inc., San Diego, CA, USA) at Centogene (Rostock, Germany)). While biallelic *WARS2* variants were identified, they were not considered to be disease-causing due to the pathogenicity classification as a variant of uncertain significance (VUS) of one variant and the poor match of phenotype between our patient and previous descriptions in the literature. The re-evaluation of the exome data and thus also of the biallelic *WARS2* variants was performed in 2021 as part of the SolveRD project (https://solve-rd.eu/) [15,16] using the GPAP (Genome-Phenome Analysis Platform; https://platform.rd-connect.eu) [17]. Newly available information from the literature in terms of variant interpretation (based on ACMG recommendations using VarSome [18] and Franklin (https://franklin.genoox.com, last accessed on 1 March 2023) and phenotypic spectrum (via PubMed (https://pubmed.ncbi.nlm.nih.gov, accessed on 01 March 2023) and OMIM (https://omim.org, accessed on 1 March 2023) was considered in this process.

Based on the now likely disease-causing role of the two *WARS2* variants in P1, we searched for additional patients with two *WARS2* variants on GPAP, which contained >10.000 exome and genome datasets of patients with rare neurological, neuromuscular, neurodevelopmental, and cancer syndromes as well as their healthy relatives [15]. Of the 10 patients identified to have two *WARS2* variants (date: 8 April 2021), submitters of patients with the p.Trp13Gly (NM_015836.4, c.37T>G) variant and a second variant at least classified as VUS were contacted, leading to the inclusion of Patient P2 and Patient P3 in this study. There were no patients with two variants classified as (likely) pathogenic.

One additional patient (P4) was included in the study (by A.T.S.A.) based on the identification of biallelic *WARS2* variants in a diagnostic setting.

The pathogenicity of these additional variants was evaluated following ACMG recommendations including the calculation of the CADD scores [19] and the prediction of the software tool MutationTaster [20].

### 2.2. Collection of Biosamples

Blood samples for Sanger sequencing were available for all four patients and their parents. Additional biosamples for further functional studies were available in three families, as follows: in Family 1, fresh blood for RNA extraction and fibroblasts cultured from skin biopsy from P1 as well as from both parents (M1 and F1); in Family 2, fresh blood for RNA extraction and fibroblasts cultured from a skin biopsy for P2; in Family 3, fresh blood samples for RNA extraction from the patient (P3) and the parents (M3 and F3). The fibroblast cell lines were cultured in Dulbecco’s modified Eagle’s medium (DMEM, Thermo Scientific, Waltham, MA, USA) supplemented with 10% fetal bovine serum (Life Technologies) and 1% penicillin/streptomycin (Life Technologies) at 37 °C and 5% CO_2_ in a humidified atmosphere. RNA was extracted from the blood samples (PAX tube system) and fibroblasts using a QIAmp RNA Extraction Kit (QIAGEN, Germantown, MD, USA) in Family 1 and Patient 2, while in Family 3, Tempus™ tubes and a Tempus™ Spin RNA Isolation Kit (Invitrogen™/Thermo Fisher Scientific, Waltham, MA, USA) was used.

### 2.3. Sanger Sequencing and Sequencing of cDNA

Sanger sequencing was performed to validate variants as well as compound heterogenicity in all four patients and parents at the referring centers (P1: Lübeck, Germany; P2: Barcelona, Spain; P3: Ljubljana, Slovenia; P4: Murcia, Spain).

To evaluate the consequences of the splice site variant in Family 3, the complementary DNA (cDNA) was synthesized by reverse transcriptase using Oligo-dT-Nucleotides of the Maxima First Strand cDNA Synthesis Kit (ThermoFisher, Waltham, MA, USA) as primers. PCR was conducted (WARS2_c.349-1G>A_F: CGGGCACTTCATAAGGGAT; WARS2_c.349-1G>A_R: TTACCTTCTTCATGGATGTGAG) and the product Sanger sequenced.

### 2.4. Staining of the Mitochondrial Network

The mitochondrial network was investigated as previously described [21,22]. Cells were either stained under basal conditions or after treatment with paraquat (2 mM) for 5 h. Subsequently, the mitochondrial network in the fibroblasts was stained with an anti-GRP75 antibody (1:1000, Abcam, Cambridge, MA, USA) in combination with the Zenon immunolabelling kit (Invitrogen, Carlsbad, CA, USA) according to the manufacturer’s protocol.

### 2.5. Western Blotting of MT-CO2

Cell pellets were extracted using RIPA extraction buffer (25 mM Tris–HCl pH 7.6, 150 mM NaCl, 1% DOC, 1% NP-40, 0.1% SDS and proteinase inhibitor cocktail with completeTM Mini and phosStopTM (both Roche), 1 tablet each for 10mL). Gels were blotted onto nitrocellulose membranes. The antibodies anti-MT-CO2 (1:5000; abcam; ab79393), Anti-WARS2 (1:1000; Sigma, St. Louis, MO, USA; SAB1408661 and 1:500, 1:1000; Invitrogen; PA5-43808), and anti-β-actin (1:2000; Sigma; A5316) for immunoblotting were used. For quantification of the Western blots, Image Lab 6.1 (Bio-Rad) was used to analyze the intensity and volume of the bands.

## 3. Results

### 3.1. Case Reports

#### 3.1.1. Patient 1

The now 20-year-old male patient (P1) was born after an unremarkable pregnancy without complications as the first and only child of healthy, non-consanguineous German parents. Due to bilateral hip dysplasia, the patient underwent a 6-week straddle pant treatment. There were initial feeding problems due to uncoordinated tongue movements; further bottle feeding was problem-free. A mild tonus dysregulation was described at the age of 4 months. At this time, a fine-beat head tremor developed. An EEG examination was performed under suspicion of epilepsy and was normal. In the fourth year of life, an increased left-sided cramping during voluntary movements was observed. There was slight intellectual disability. From the age of 6 years, tremor of both hands appeared, which was slowly progressive in the course. Propranolol therapy showed no effect. The tremor was progressive, slight at rest, and clearly increasing as intention tremor. Over the next years, though, the tremor considerably deteriorated and the patient also developed gait instability with small steps and dystonia. The cognitive function showed mild intelligence impairment. He suffered from anxiety, which affected his daily life at around the age of 13 years. Later, the anxiety became less prominent, with an uneasy feeling in larger open spaces.

At the age of 19 years, he presented with marked Holmes Tremor, generalized dystonia, and leg spasticity with increased reflexes. A video of the examination can be found in the Supplementary Materials (Supplementary Video S1). Treatment with levodopa was established at the age of 15 years, improving tremor and gait difficulties. Later, biperiden was added to the medication, which led to further improvement of the tremor and the ability to move.

Brain MRI was unremarkable. Cerebrospinal fluid examination showed normal lactate, reduced homovanillic acid, and slightly reduced tetrahydrobiopterin and alanine (Table 1).

#### 3.1.2. Patient 2

The now 20-year-old male patient (P2) was born after an unremarkable pregnancy without complications as the only child of healthy, non-consanguineous Spanish parents. He walked independently at 22 months and was noted to be clumsy from early on. Language was also delayed and with morphosyntactic deficit. He needed special support from primary school. He was referred at age 9 years, when he displayed mild intellectual disability, clumsiness, and some parkinsonian features, including bradykinesia, cogwheel rigidity, and resting and postural tremor. The tremor worsened during emotional upset up to the point of making him fall to the ground. Over the years, some additional features appreciated during follow-up were slow and hypometric saccadic ocular movements, cervical and upper limb dystonia, lower limb spasticity, and occasional action myoclonic jerks. Treatment with levodopa improved both tremor and bradykinesia, with functional benefit in gait and manipulative skills, and prevented further episodes of falling.

A DaTScan showed the marked loss of tracer uptake bilaterally over the putamina and moderate decrease on the right caudate nucleus. There was also a suggestion of increased extrastriatal cortical activity.

**Figure 1:**
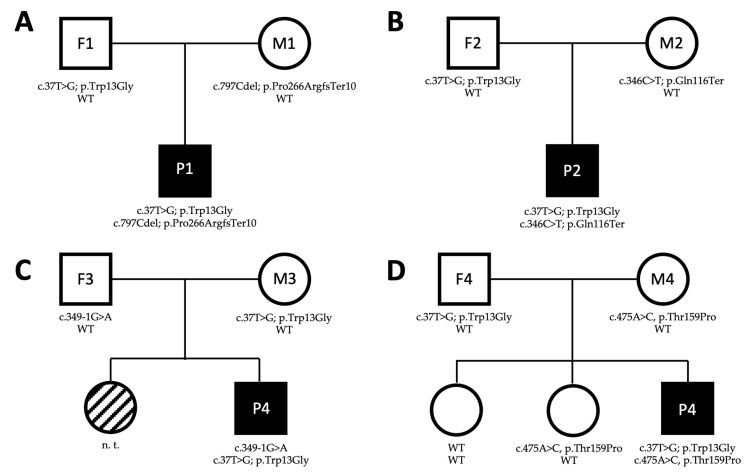
Pedigree of the family of Patient 1 (**A**), Patient 2 (**B**), Patient 3 (**C**), and Patient 4 (**D**). WT: wildtype, n.t.: not tested; square: male, circle: female, white: unaffected, black: affected, striped: isolated tremor.

Serial brain MRI showed the mild prominence of the cerebellar folia plus pallidal T2WI hypointensity at the last examination. The patient–parent trio exome sequencing in 2018, and re-analysis in 2021, revealed compound heterozygous variants in *WARS2* with a maternally inherited truncating variant (NM_015836.4: c.346C>T; p.Gln116Ter) and a paternally inherited missense variant (NM_015836.4: c.37T>G; p.Trp13Gly) (Figure 1B).

#### 3.1.3. Patient 3

The now 38-year-old male patient (P3) was born as the second child to non-consanguineous Slovenian parents. Around the age of 13 years, the patient presented with tremor in the upper extremities along with occasional myoclonic jerks. The tremor first presented on the left body side, but slowly progressed to bilateral resting tremor. His symptoms gradually progressed until, at the age of 25 years, he first visited a neurologist and was diagnosed with early-onset Parkinson’s disease. Besides prominent hand tremor, he presented with hypomimia, bradykinesia, mild dysarthria, stiffness, and mild dystonic posturing of the left leg during walking. The laboratory findings were unrevealing and brain MRI was normal, but DaTScan imaging showed reduced tracer uptake bilaterally over the putamina. Neuropsychological testing at the age of 33 years revealed mild cognitive impairment as well as an introverted, unsociable character with a lack of trust. The patient and his parents also reported mood changes, anxiety, and occasional irritability. Treatment with rasagiline, levodopa, and entacapone was established and the tremor, bradykinesia, and rigidity improved. The 5-year older sister was reported to have tremor since childhood; however, no examination was performed.

A patient–parent trio exome sequencing in 2022 revealed compound heterozygous variants in *WARS2* with a paternally inherited splice site variant (NM_015836.4: c.349-1G>A) and a maternally inherited missense variant (NM_015836.4: c.37T>G; p.Trp13Gly) (Figure 1C).

#### 3.1.4. Patient 4

The now 2-year-old male patient (P4) was born at 34 weeks after an uneventful pregnancy as the third child of healthy, non-consanguineous parents of Moroccan origin. His developmental milestones, predominantly gross and fine motor skills, were delayed from the early stages. At 15 months of age, he developed generalized gross resting tremor that started in the lower limbs and then progressed to the upper limbs and head. This tremor is intensified by voluntary actions. He does not have myoclonus or dystonia. He also has developed lower limb hypertonia and Babinski sign.

EEG, brain MRI, abdominal ultrasound, and ophthalmological examination were normal. Screening for metabolic disorders and ArrayCGH were also normal. The patient’s motor skills and tremor improved with levodopa treatment.

A patient–parent trio exome sequencing in 2022 revealed compound heterozygous variants in *WARS2* with a maternally inherited missense variant (NM_015836.4: c.475A>C, p.Thr159Pro) and a paternally inherited missense variant (NM_015836.4: c.37T>G; p.Trp13Gly) (Figure 1D).

### 3.2. Characterization of WARS2 Variants

In all four patients, the same missense variant (NM_001378227.1: c.37T>G; p.Trp13Gly) was found. According to the ACMG recommendations, this variant is classified as pathogenic due to the criteria PM3, PP5, and PS3, it has an allele frequency on gnomAD of 0.33% overall (and 0.46% in non-Finnish Europeans), and has conflicting interpretation of pathogenicity on ClinVar (however with 4/5, classifying the variant as pathogenic or likely pathogenic). The CADD score for the variant is 22.7, and half of all previously published cases (12/24; Table 2) of *WARS2*-related disorders carry this variant (with the four new cases, it is 16/28, 57.1%; Table 2). Functional studies showed protein mislocalization due to the variant [3].

P1 and P2 carry an additional truncating variant. The c.797delC variant of P1, resulting in a change of Proline to Arginine at position 266 with termination of the amino acid sequence at position 276, is classified as pathogenic according to ACMG and was previously published in four patients. The c.346C>T variant of P2 is classified as likely pathogenic and was not previously described. This variant results in the termination of the amino acid sequence at position 116.

**Figure 2:**
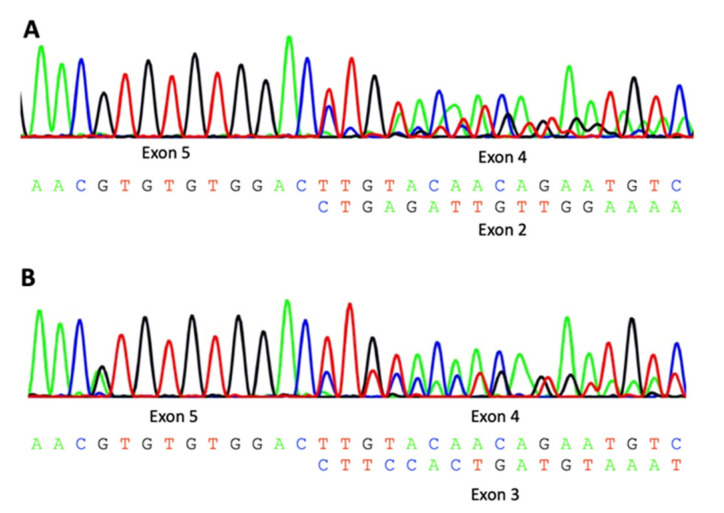
cDNA sequencing of P3 (**A**) and M3 ((**B**); does not carry the c.349-1G>A variant) with skipping of exon 3 and 4 in P3 and skipping of exon 4 only in M3 (alternative transcript, NM_001378231.1).

The novel splice site variant c.349-1G>A in P3 is classified as likely pathogenic and the sequencing of cDNA showed the skipping of exon 3 and 4 in half of the transcripts (Figure 2).

In P4, the novel second missense variant c.475A>C is classified as VUS, but with a CADD score of 24, is not listed in gnomAD and is predicted to be disease causing by MutationTaster.

Based on the compound heterozygous state of the detected variants and the clinical presentation in line with the previously described phenotypes (Table 1), a diagnosis of *WARS2*-releated disorder was made in all four patients.

### 3.3. Mitochondrial Integrity

**Figure 3:**
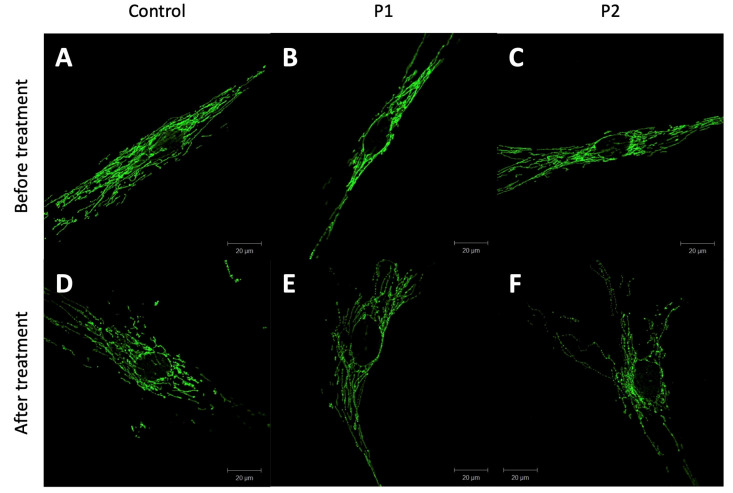
Representative pictures of immunostaining of cultured fibroblasts with GRP75 of a control (**A**,**D**), P1 (**B**,**E**), and P2 (**C**,**F**) before (**A**–**C**) and after paraquat treatment (**D**–**F**).

After staining the mitochondrial network in the cultured fibroblasts of controls as well as of P1 and P2 with anti-GRP75, the dissolvement of mitochondrial integrity and mitochondrial branching after paraquat treatment was more pronounced in the patients’ fibroblasts (Figure 3). 

### 3.4. Respiratory Chain and WARS2 Level

**Figure 4:**
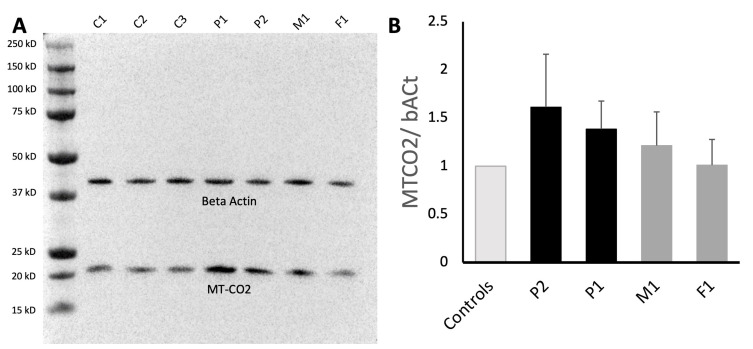
Analysis of the mitochondrially encoded Cytochrome C Oxidase II protein. (**A**) Representative Western blot for three controls (C1–C3), two biallelic patients (P1, P2), and two heterozygous unaffected relatives (M1, F1) showing protein levels of MT-CO2, which is increased in the patients and in M1 when compared to the controls. Beta-actin was stained as a control for equal loading. (**B**) Quantification of the signals from three Western blots was performed by analyzing the intensity and volume of the bands using Image Lab 6.1 (Bio-Rad). Ratio of MT-CO2 and β-actin was calculated first, followed by the mean value of all controls, which was standardized as 1. Mean values of patients (P1 and P2) and relatives (M1 and F1) are given as ratio to controls. Error bars: standard deviation. Light gray: wild type; dark gray: heterozygous carriers (parents); black: biallelic carriers (patients).

A Western blot of the mitochondrially encoded Cytochrome C Oxidase II (MT-CO2) as part of the complex IV of the reparatory chain showed an increased expression of MT-CO2 in P1 and P2 compared to the controls, as well as a less pronounced increase in M1 with the truncating variant and normal values in F1 with the missense change (Figure 4). Western blots with the anti-WARS2 antibodies only showed unspecific bands without a clear band at the expected level of 24.8 kD (see Supplementary Figure S1) and thus could not be analyzed.

## 4. Discussion

In this study, we present four new patients with an early-onset tremor–parkinsonism syndrome, who were identified to have a *WARS2*-related disorder. All patients carried a recurrent, quite common missense variant and responded well to levodopa. Furthermore, we show an effect on splicing of the first reported intronic splice site *WARS2* variant. We also demonstrate impaired mitochondrial integrity and alterations of the protein levels of components of the mitochondrial respiratory chain.

Previous studies showed divergent results when investigating the enzyme activity and protein levels of the different subunits of the mitochondrial chain. Complex II was normal in all biosamples (fibroblast, liver biopsy, muscle biopsy, neuroepithelial-like stem cells) in all 10 investigated patients, most likely due to the fact that complex II is entirely nuclear-encoded [4,5,6,7,8,12]. Complex I and III were reported to be reduced in one case in patient-derived fibroblasts [4], while in the liver biopsy of another patient, only complex I was reduced [8]. In all other biosamples of patients, both complex I and III were unimpaired [5,6,12]. Complex IV was the complex most often reported as reduced in the form of the reduction of the subunit MTCO2 in fibroblasts [4,5], liver biopsy [7] and neuroepithelial-like stem cells [8], but there were also reports of normal levels [5,6,12]. In our tested patients (P1 and P2), we found an increase in MTCO2 protein levels of about 50% compared to the controls, with the heterozygous parents having either normal MTCO2 levels or slightly elevated levels. This increase is most likely a compensating mechanism of the impaired mitochondrial respiratory chain. Previous studies of the enzyme activity of the respiratory chain showed a similar diversity, with normal results in fibroblasts [5,8] and muscle biopsies [6] as well as pathological results in fibroblasts [12], liver biopsies [5,7], muscle biopsies [5], and neuroepithelial-like stem cells [8]. We also investigated the mitochondrial integrity of the patient-derived fibroblasts and found an altered mitochondrial network with reduced mitochondrial branching as a sign of impaired mitochondrial integrity caused by pathogenic *WARS2* variants. Overall, further functional studies in *WARS2* are needed to determine the exact mechanism of mitochondrial impairment and the effects on mitochondrial function. For this, the generation of specific WARS2 antibodies to detect the protein on the endogenous level is warranted.

Since the original reports of the first *WARS2* patients in 2017 with predominant cognitive impairment [3,4,5], an increasing number of patients with a predominant movement disorder phenotype have been added to the literature [6,12,13,14], resulting in a broad phenotypical spectrum, typical for mitochondrial disorders. While cognitive impairment is still the most commonly reported feature (in 67% of all patients), dystonia (at least 50%), tremor (at least 38%), and parkinsonism (at least 21%) are often present in patients with *WARS2*-related disorder. However, the clinical data available are still scarce. Our four patients all had a predominant movement disorder phenotype including tremor (4/4), parkinsonism (4/4), and dystonia (3/4), which might also be linked to the recruitment strategy (all patients were evaluated within an ERN-RND (European Reference Network for Rare Neurological Diseases) initiative with a focus on movement disorders). Notably, all patients improved with levodopa therapy. This makes *WARS2*-related disorder an important differential diagnosis in patients with early-onset dystonia, tremor, and/or parkinsonism with a good treatment option. The recent increase in reported *WARS2*-related disorder cases suggests it to be an underdiagnosed and underreported disorder.

One factor contributing to the suspected underdiagnosis of *WARS2*-related disorder is the recurrent variant NM_015836.4: c.37T>G; p.Trp13Gly, which all four of our patients carried as well as half of the previously reported patients. The p.Trp13Gly variant is reported in six homozygous carriers in gnomAD [23], which might lead to discarding this variant as a disease-causing variant if one is oblivious to its role in *WASR2*-releated disorder in the compound heterozygous state. The variant has been classified as a hypomorphic variant [13,14], a variant that is only disease causing in the case of compound heterogenicity when coupled with another disease-causing variant and not harmful in the case of homozygosity. Patients carrying this hypomorphic variant in combination with a missense variant seem to have a milder form [13] with a later onset (mean 4 years compared to <1 year) in most cases, with patient P4 being one, but not the only exception [12,14]. Awareness of this specific variant therefore is important to diagnose patients with *WARS2*-related disease.

## 5. Conclusions

In summary, we report four patients with an early-onset tremor–parkinsonism syndrome due to biallelic *WARS2* variants. All patients carried the same missense variant on one allele and responded well to levodopa.

## Figures and Tables

**Table 1:** Clinical description of the newly described patients and patients in the literature.

Category	P1	P2	P3	P4	Literature (n = 24) ^1^ [3,4,5,6,7,8,9,10,11,12,13,14]
Sex	Male	Male	Male	Male	Male 11/24 (45.8%)
Age of onset	0.5 y	9 y	13 y	1 y	Mean 2.0 y [range 0–12 y]
Age at last examination	20 y	20 y	38 y	2 y	Mean 16.4 y [range 0–52 y]
First signs and symptoms	Tremor	Tremor, intellectual disability	Tremor	Tremor, developmental delay	Hyperkinetic MD 8/22
					Developmental delay 8/22
					Others ^2^ 6/22
Tremor	yes	yes	yes	yes	9/9
Parkinsonism	yes	yes	yes	yes	5/5
Dystonia	yes	yes	yes	no	12/12
Other hyperkinetic MD ^3^	no	Myoclonic jerks	Myoclonic jerks	no	6/6
Ataxia	no	no	no	yes	8/8
Increased muscle tone	yes	yes	yes	yes	10/10
(Axial) hypotonia	no	no	no	yes	8/8
Muscular weakness	no	no	no	no	4/4
Seizures	no	no	no	no	7/10
Developmental delay	no	no	no	yes	7/7
Intellectual disorder	mild	mild	mild	(mild to moderate) ^4^	16/20
Mild to moderate					6/15
severe					9/15
Lactate acidosis	no	no	no	no	3/4
L-Dopa response	good	good	good	good	6/8
MRI abnormal	no	Pallidal T2 hypointensity	no	no	5/10 ^5^
DaTScan abnormal	n. a.	Yes	yes	n. a.	3/3

^1^ Not all symptoms were reported in all patients. Number of patients with information on specific symptoms is indicted behind the slash. ^2^ Lactate acidosis, muscular hypotonia, dystonia, seizures. ^3^ myoclonus/myoclonic jerks, chorea/choreatic movements, ballism. ^4^ Difficult to evaluate due to young age of patient. ^5^ Cerebellar atrophy, leukoencephalopathy, thin corpus callosum. MD: movement disorder. The family history for neurological disorders was unremarkable. Candidate gene genetic testing including *ATP13A2*, *C19orf12*, *COASY*, *CP*, *DCAF17*, *FA2H*, *FTL*, *GTPBP2*, *IBA57*, *PANK2*, *PLA2G6*, and *WDR45* did not reveal a disease-causing variant in 2017. A parent–patient trio exome sequencing in early 2018 with re-analysis in 2021 revealed compound heterozygous variants in *WARS2* with a maternally inherited truncating variant (NM_015836.4: c.797Cdel; p.Pro266ArgfsTer10) and a paternally inherited missense variant (NM_015836.4: c.37T>G; p.Trp13Gly) (Figure 1A).

**Table 2:** WARS2 variants of the newly described patients and patients in the literature.

Mutation		Patients		Families	
c.622G>T	p.Glu208Ter	1	3.6%	1	4.3%
c.1054G>A	p.Glu352Lys	1	3.6%	1	4.3%
c.134G>T	p.Gly45Val	1	3.6%	1	4.3%
c.231C>G	p.His77Gln	1	3.6%	1	4.3%
c.487C>T	p.Leu163Phe	1	3.6%	1	4.3%
c.679A>G	p.Met227Val	1	3.6%	1	4.3%
c.683C>G	p.Ser228Trp	1	3.6%	1	4.3%
c.532G>C	p.Val178Leu	1 ^#^	3.6%	1	4.3%
c.1045G>A	p.Val349Leu	1	3.6%	1	4.3%
**c.346C>T**	**p.Gln116Ter**	1	3.6%	1	4.3%
**c.349-1G>A**	**Splice alteration**	1	3.6%	1	4.3%
**c.475A>C**	**p.Thr159Pro**	1	3.6%	1	4.3%
c.149G>A	p.Gly50Asp	2	7.1%	2	8.7%
c.325delA	p.Ser109Alafs*159	2	7.1%	1	4.3%
c.298_300delCTT	p.Leu100del	3	10.7%	2	8.7%
c.91-8725_348+27116del36099	p.Lys31_Gln116del	3	10.7%	2	8.7%
c.833T>G	p.Val278Gly	3	10.7%	2	8.7%
**c.797delC**	p.Pro266Argfs*10	5	17.9%	3	13.0%
c.938A>T	p.Lys313Met	9	32.1%	7	30.4%
**c.37T>G**	**p.Trp13Gly**	16	57.1%	9	39.1%

Variants in bold are carried by patients reported in this paper. ^#^ Patient is homozygous for this variant.

## Data Availability

The raw data supporting the conclusions of this article will be made available by the authors upon reasonable request.

## Supplementary Materials

The following supporting information can be downloaded at: https://www.mdpi.com/article/10.3390/genes14040822/s1, Video S1: Examination of P1 Deskription: Examination of P1 at the age of 19 years showing voice tremor, rest tremor, postural tremor and action tremor as well as generalized dystonia. Figure S1: Representative Western blot for three controls (C1–C3), two biallelic patients (P1, P2), and two heterozygous unaffected relatives (M1, F1) showing unspecific bands without a clear band at the expected level of 24.8 kD. Beta-actin was stained as a control for equal loading.
