# Supplementary material for: The Expanding Phenotypical Spectrum of WARS2-Related Disorder: Four Novel Cases with a Common Recurrent Variant
Source: Genes (Basel). 2023 Mar 29;14(4):822. doi: 10.3390/genes14040822 (PMC10137540; doi:10.3390/genes14040822)
Supplement: Supplementary file 1 [file genes-14-00822-s001.zip › genes-2303163-supplementary/Supplementary Figure S1.docx]

**
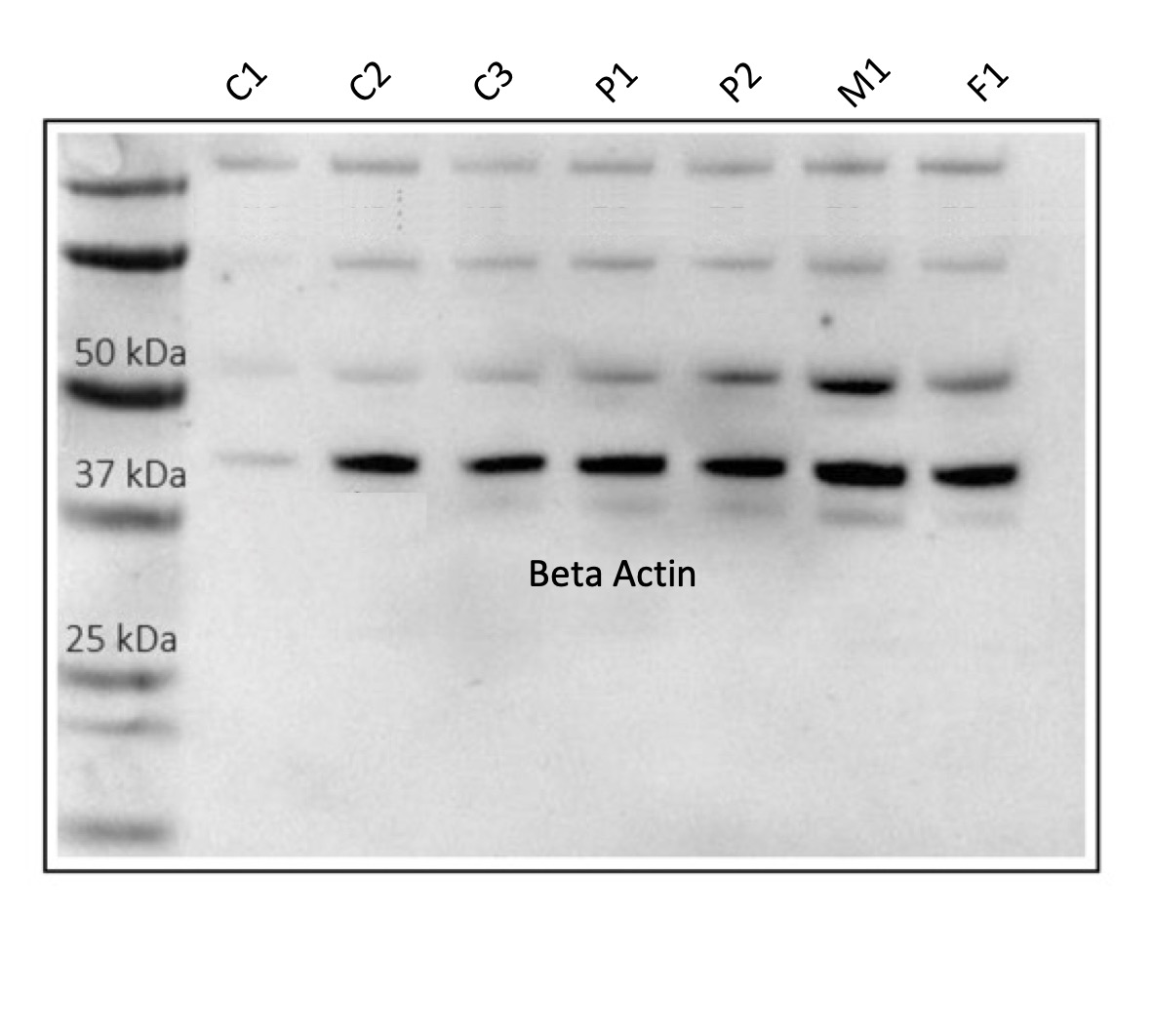
Figure S1**. Representative Western blot for three controls (C1–C3), two biallelic patients (P1, P2), and two heterozygous unaffected relatives (M1, F1) showing unspecific bands without a clear band at the expected level of 24.8 kD. Beta-actin was stained as a control for equal loading.
